# Local Versus Systemic Antibiotics for Diabetic Foot Infection—A Systematic Review and Meta‐Analysis

**DOI:** 10.1111/1753-0407.70201

**Published:** 2026-03-10

**Authors:** Anas Khan, Hester Lacey, Helena Michels, Mohamed Elahwal, Bjorn Telgenkamp

**Affiliations:** ^1^ University Hospitals Sussex NHS Foundation Trust, Eastern Rd, Brighton and Hove Brighton UK; ^2^ Ashford and St. Peter's Hospitals NHS Foundation Trust Chertsey UK; ^3^ Flinders University Bedford Park South Australia Australia; ^4^ University of Sussex Brighton UK

**Keywords:** antibiotics, diabetic foot infection, meta‐analysis, osteomyelitis

## Abstract

Diabetic foot infection (DFI) includes soft tissue infection and osteomyelitis below the ankle and is a leading cause of lower limb amputation and mortality in diabetic patients. Treatment involves prolonged antibiotic therapy with surgical debridement or amputation. Local antimicrobial therapy offers an adjunct or alternative to systemic therapy. This systematic review and meta‐analysis assessed the efficacy of systemic antibiotic therapy compared with local and combination (local and systemic) therapy in DFI. A meta‐analysis was conducted according to the Preferred Reporting Items for Systematic Reviews and Meta‐Analysis (PRISMA) guidelines. A systematic search for studies was performed in eight sources (PROSPERO: CRD42024518421). The primary outcome was clinical cure rates, with secondary outcomes including clinical improvement, time to clinical cure, recurrence, amputation rates, and pathogen eradication. Twenty‐one studies with 2188 participants met the inclusion criteria, including 12 randomized controlled trials and 9 observational studies. Combination antibiotics were associated with increased clinical cure rates compared with systemic antibiotics alone (OR 2.08; 95% CI 1.30–3.35; *p* < 0.05) and faster healing times (Mean −9.8 days; 95% CI −15.1 to −4.4; *p* < 0.05); however, results failed to reach significance when looking at randomized studies alone. Results for local antibiotics alone were non‐significant for all outcomes. This meta‐analysis suggests that definitive conclusions about the use of local antibiotics in DFI are limited by bias and heterogeneity within included studies. Combined local and systemic antibiotic treatment may allow for direct tissue infiltration of antimicrobial therapy. However, high‐quality, blinded randomized controlled trials are required before routine clinical adoption.

## Highlights

Topical antibiotics are commonly used in local infections. There is limited guidance on their use in diabetic foot infection (DFI). This systematic review summarizes the existing evidence relating to local antibiotics in DFI in improving rates of clinical cure, clinical improvement, antimicrobial eradication, infection recurrence, and amputation rates in DFI, alone and in combination with systemic therapy. This study suggests the use of combination antibiotic therapy may improve cure rates and speed healing times in DFI. However, this review supports the need for further prospective, blinded, randomized studies before the routine use of local antibiotics can be routinely recommended.

## Graphical abstract

Combined local and systemic antibiotic therapy improved clinical cure rates and shortened healing time in diabetic foot infections compared with systemic therapy alone. However, due to the low quality of existing studies, high‐quality randomized controlled trials are needed to confirm these findings.

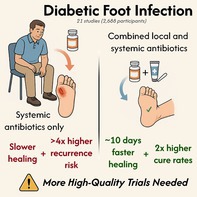

**Abbreviations**
95% CI: 95% confidence intervals
ABC: antibiotic bone cement
ABPI: ankle‐brachial pleasure index
DFI: Diabetic foot infection
DFO: diabetic foot osteomyelitis
DFU: diabetic foot ulcer
MDR: multidrug resistance
OR: odds ratio
PICO: Population, intervention, comparison, and outcome
PRISMA: Preferred Reporting Items for Systematic Reviews and Meta‐Analysis
RCTs: randomized controlled trials
TTT: tibia transverse transport
WIfI: Wound, Ischemia, and Foot Infection

## 1 Introduction

Diabetic foot infection (DFI) describes the development of soft tissue infection or osteomyelitis below the ankle in patients with diabetes mellitus [1]. DFI manifests with cellulitis, ulceration, and tissue loss, and can progress to invasive bone infection [1]. DFI remains a significant cause of morbidity and mortality in the diabetic population, with a lifetime risk estimated at 19%–34% and conferring an increased risk of complications including prolonged hospitalization, limb amputation (up to 20% of cases), and a 2.5‐fold increase in mortality compared with those who do not develop DFI [2, 3, 4].

Management of DFI includes adequate wound care, pressure area offloading, tight glycaemic control, debridement of necrotic tissue, revascularisation, and antibiotic therapy [5]. Prolonged systemic antibiotics tailored to wound and blood culture results are typically required [6]. Failure of antibiotic therapy resulting in spread of infection to involve bone or joint may necessitate debridement to the level of clean tissue and risks subsequent need for limb amputation [2, 3]. The likelihood of failure is increased by worsening microvascular ischaemia and neuropathy, regional trauma, and development of multidrug resistance (MDR) organisms [5]. Infection is often polymicrobial, increasing the challenge of determining appropriate antibiotic therapy and increases the risk of MDR development [7].

Local agents, including antiseptic dressings (e.g., silver, cadexomer iodine and hypochlorous solutions) and topical antibacterials (e.g., honey) have been associated with reduced antimicrobial load in infected wounds and are commonly used in DFI, despite limited definitive evidence for their benefit [7]. Local antibiotic interventions (powders, beads and impregnated cement) are less commonly used in diabetic foot osteomyelitis (DFO). Indeed, there is limited high‐quality evidence for their benefit, with use often determined by clinician or centre preference [8]. Topical antibiotic therapy can achieve high tissue drug concentrations, with limited systemic absorption, reducing the risk of MDR and adverse systematic effects of antibiotic therapy [9]. Definitive evidence for benefit of local antibiotics in DFI remains limited by lack of clinical studies, with existing evidence predominantly in non‐human tissue models [3]. Administration of local antibiotics alone or in combination with systemic therapy has potential to improve healing times and recovery rates in DFI, reducing the risk of disease progression and associated complications [7].

This paper systematically reviews the available literature comparing local versus systemic antibiotics in DFI, evaluates their benefit in addition to or in place of systemic therapy, and supports establishment of regional and national guidelines relating to their use.

## 2 Methods

### 2.1 Search Strategy and Information Sources

A systematic literature search was performed combining key terms such as “systemic”, “local”, “antibiotics” and “diabetic foot infection” and various synonyms in differing combinations. These search terms were developed in collaboration with medical librarians to ensure the highest capture rate. The full search strategy is described in Supplement 1 and was performed on 1st September 2024. This search was entered into 7 databases: The Cochrane Library, MEDLINE, EMBASE, Emcare, Global Health, Web of Science and Scopus, and 1 gray literature source: Google Scholar. The reference lists of key papers were reviewed to further identify articles of interest. This review is reported using Preferred Reporting Items for Systematic Reviews and Meta‐Analysis (PRISMA) guidelines [10], and was prospectively registered in the international database of prospectively registered systematic reviews in health and social care (PROSPERO)—CRD42024518421 [11].

### 2.2 Eligibility Criteria and Selection Process

Articles were included if they reported on either local or combination therapy (local plus systemic antibiotics) versus systemic antibiotics alone in patients with DFI, diabetic foot ulcer (DFU) or DFO for any severity of disease (as graded by University of Texas Staging System for Diabetic Foot Ulcers, Wagner Grading, International Working Group on the Diabetic Foot or any other appropriate system). Systemic therapy included oral and intravenous routes of delivery. Patients were required to be adults, have diabetes and have a lesion below the ankle. Patients with concomitant vascular disease were excluded unless urgent revascularisation was performed prior to requirement. Both inpatient and outpatient treatment were included. Preceding surgery (e.g., drainage, debridement, or minor amputations) was also allowed. Single arm studies, letters, reviews, abstracts, and case series were also excluded. Full inclusion and exclusion criteria are described in Supplement 2.

### 2.3 Data Items

The outcomes of interest are described and defined below:

- **Clinical cure**—resolution of signs and symptoms of infection and healing of a wound. This was the primary outcome.
- **Time to clinical cure**—Time taken in days for clinical cure
- **Clinical improvement**—decrease in the clinical features of inflammation (e.g., exudate, warmth, erythema, induration, tenderness, swelling) but without complete wound healing.
- **Complete microbiological eradication**—involved sending tissue or fluid for culture before initiation of treatment or intraoperatively and sending sample for culture at the end of treatment or final follow up; no bacterial growth on the post treatment sample was considered successful.
- **Infection recurrence**—a previously cured or improved wound which later showed worsening signs of infection by final follow up. It could also include a new focus of infection at or adjacent to the previous site of infection within the follow up period.
- **Amputation/further intervention**—a patient requiring admission and further debridement or major or minor amputation during follow up period.

### 2.4 Data Collection Process

Title and abstract screening and full‐text review were performed by two separate reviewers (AK, HL) with reference to study inclusion and exclusion criteria. Conflicts were resolved by consensus and where agreement could not be reached a third reviewer's opinion was sought (HM). Data collection was performed using a bespoke data collection tool on the Covidence platform [12]. Two reviewers collected data separately (AK, HL), with conflicts resolved by consensus (HM).

### 2.5 Statistical Analysis and Effect Measures

This study stratified by soft tissue disease or presence of bone disease. Meta‐analysis was performed in R software using the meta package with effects presented as odds ratio (OR) with 95% confidence intervals (95% CI), unless otherwise specified [13]. ORs were pooled with the random‐effects model using the Mantel–Haenszel test. Meta‐analyses performed included an assessment of heterogeneity of included studies through calculation of *I* ^2^, 75% or greater equated to considerable heterogeneity as per Cochrane handbook guidance [14]. Subgroup analysis included soft tissue and osteomyelitis disease. Sensitivity analysis for each outcome including only RCTs was performed.

### 2.6 Risk of Bias and Quality Assessment

The risk‐of‐bias (RoB2) tool was used for assessing randomized trials, whilst the Risk of Bias In Non‐randomized Studies—of Interventions (ROBINS‐I) tool was used for non‐randomized studies [15]. Additionally, a summary of findings table was created, and the quality of evidence was graded using the Grading of Recommendations Assessment, Development and Evaluation (GRADE) system, with evaluations performed through an online application (https://gdt.gradepro.org/app).

## 3 Results

**FIGURE 1:**
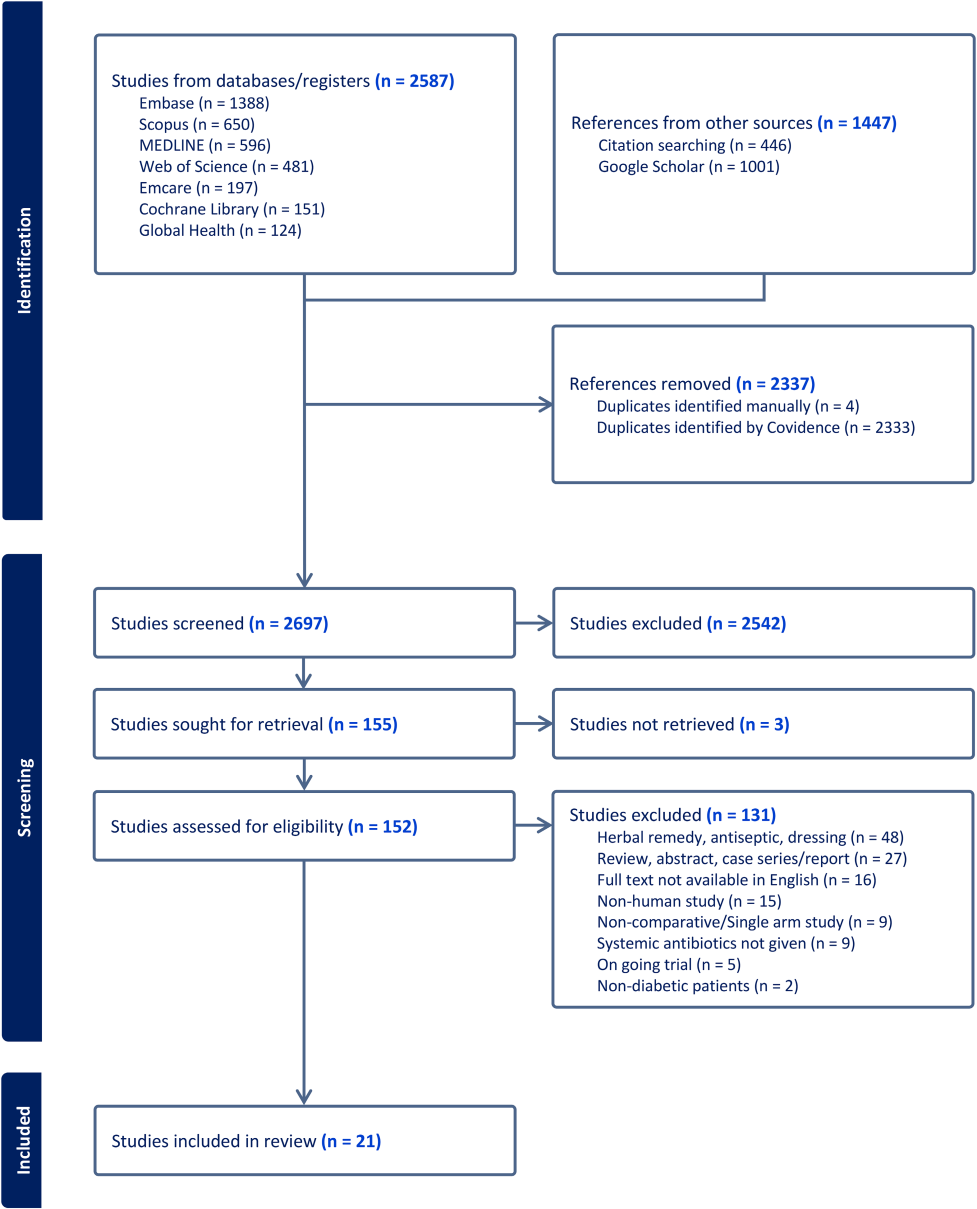
Preferred Reporting Items for Systematic Reviews and Meta‐Analyses (PRISMA) flow diagram for this systematic review.

After elimination of duplicates, 2697 articles were screened by title and abstract. Full texts for 3 references could not be retrieved via authors' organization or the British Library [16, 17, 18]. This produced 152 studies for full text review, of which 21 met the inclusion criteria totalling 2688 participants [Figure 1]. The largest had 835 participants [19], whilst the smallest had 25 [20]. There were 12 RCTs, of which 2 were unpublished trial data [19, 21, 22, 23, 24, 25, 26, 27, 28, 29, 30, 31], 8 retrospective observational studies [20, 32, 33, 34, 35, 36, 37, 38] and 1 prospective observational study [39]. Countries of paper origin included USA [19, 23, 24, 25, 26, 28, 34], China [27, 33, 36, 37], Italy [30, 32, 38], Australia [35], Switzerland [22], Greece [20], Iraq [21], Prague [31], Mexico [29], and Pakistan [39]. Mean pooled age was 61 years and 68% of participants were male. Preceding debridement prior to antibiotic therapy was performed regularly in all but two studies (it was not compulsory in Martinez‐De Jesus et al. study and Jones et al. did not provide preceding treatment information) [23, 29].

**FIGURE 2:**
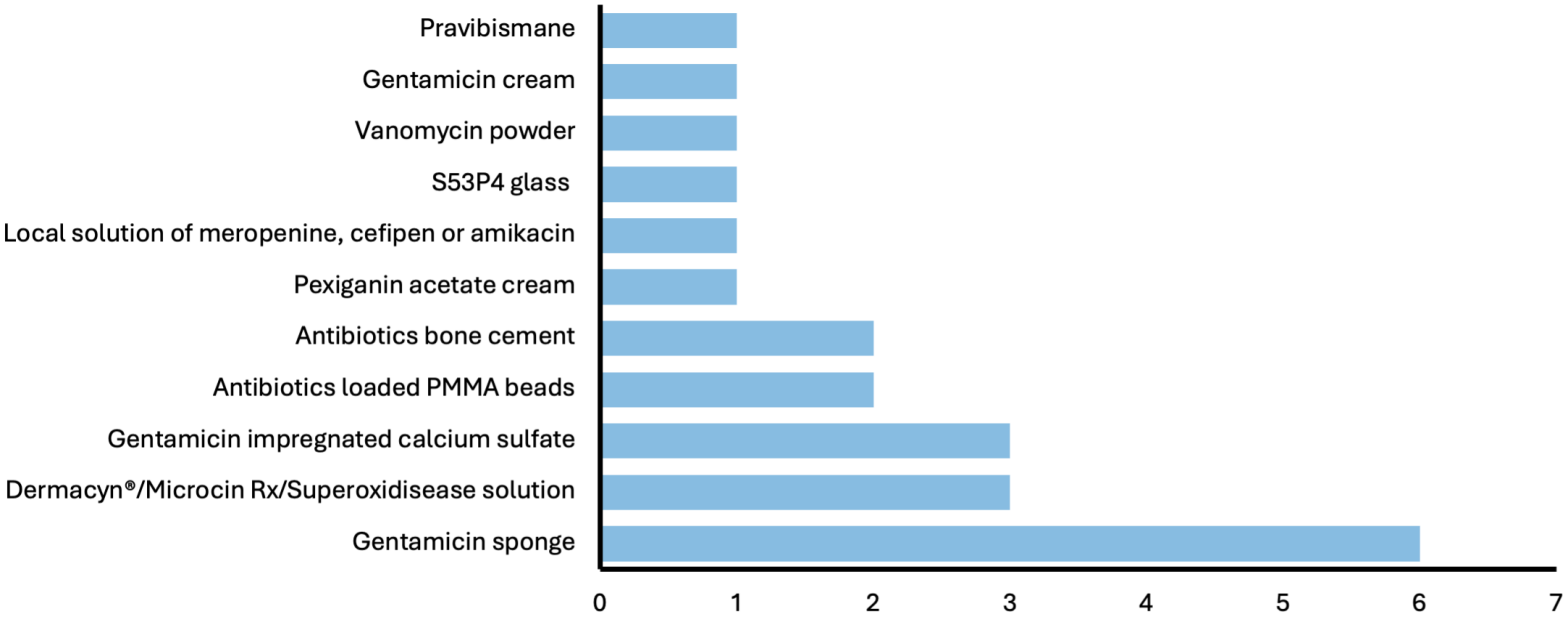
Bar chart showing frequency of local agents studied. PMMA, polymethylmethacrylate.

There was significant heterogeneity between studies, in terms of severity of disease, type of topical formulations and individual antimicrobial drug/compound [Table 1]. Studies either looked at soft tissue disease only [19, 21, 22, 23, 24, 26, 39] or those with bone infection (osteomyelitis) [20, 25, 27, 28, 29, 30, 31, 32, 33, 34, 35, 36, 37, 38]. Three studies compared local and systemic antimicrobials [19, 21, 26], seventeen studies compared local combined with systemic (i.e., combination therapy) and systemic alone [20, 22, 23, 25, 27, 28, 29, 30, 31, 32, 33, 34, 35, 36, 37, 38, 39], and one study performed a 3‐arm trial comparing local alone, combination and systemic alone [24]. Length of treatment varied between 7 and 56 days, as did follow‐up (7–540 days). Gentamicin was most commonly used local antimicrobial and came in collagen sponge [22, 23, 25, 26, 31], cream [39], bead [20] and cement forms [27, 33, 35, 36, 37, 38]. The other topical antimicrobials included pexiganan acetate cream [19], pravibismane [28], topical super oxidized solutions (Microcin Rx, Dermacyn) [24, 29, 30], meropenem solution [21], vancomycin powder [34], and S53P4 glass [Figure 2] [32]. All studies adjusted antibiotic therapy once microbiological culture and sensitivities were available.

**TABLE 1:** Summary of studies included in the meta‐analysis.

Study and Country	Funding	Study type	Disease	Severity	Sample (*n*)	Age (yrs)	Male (%)	Length of treatment (days)	Summary	Intervention drug(s)	Comparator drug(s)	Outcomes
Prior et al., 2007; USA	Innocoll	RCT	Soft tissue only	Mild	69	59	41	?21–35	Local vs. systemic	Gentamycin‐collagen sponge OD	Levofloxacin 750 mg PO OD	Clinical cure; Clinical improvement; Complete microbiological eradication from baseline
Martinez‐De Jesus et al., 2007; Mexico	None	RCT	Osteomylitis	NR	37	64	47	≥ 10	Local & systemic vs. systemic	Superoxidised solution bi/weekly with pentoxyphylline 1.2 g/day	Pentoxyphylline 1.2 g/day	Clinical improvement
Lipsky et al., 2008; USA	Magainin Pharmaceuticals and SmithKline Beecham	RCT	Soft tissue only	Mild/Moderate	835	60	68	14–28	Local vs. systemic	Pexiganin acetate cream 1/2% BD	Ofloxacin 200 mg BD	Clinical improvement; Reinfection rates; Amputation rates; Complete microbiological eradication from baseline
Piaggesi et al., 2010; Italy	Oculus Innovative Sciences	RCT	Osteomylitis	Moderate/Severe	40	62	NR	180	Local & systemic vs. systemic	Topical Dermacyn Wound Care 5‐20 mL OD with systemic piperacillin/tazobactam and metronidazole ± teicoplanin	Piperacillin/tazobactam and metronidazole ± teicoplanin	Clinical cure; Reinfection rates; Amputation rates
Landsman et al., 2011; USA	Oculus Innovative Sciences	RCT	Soft tissue only	Mild	66	57	73	9–11	Local vs. local & systemic vs. systemic	Topical Microcin Rx 25 mL OD; Topical Microcin Rx OD with levofloxacin 750 mg PO OD	Levofloxacin 750 mg PO OD	Clinical cure; Clinical improvement
Lipsky et al., 2012; USA	Innocoll	RCT	Osteomylitis	Moderate	56	57	68	7–28	Local & systemic vs. systemic	Gentamycin‐collagen sponge 32.5 mg or 130 mg OD with levofloxacin 750 mg PO OD	Levofloxacin 750 mg PO/IV OD	Clinical cure; Complete microbiological eradication from baseline
Varga et al., 2014; Prague	None	RCT	Osteomylitis	NR	45	62	73	NR	Local & systemic vs. systemic	Gentamicin‐impregnated collagen sponge with systemic antibiotics (NR)	Systemic antibiotics (NR)	Time to clinical cure; Amputation rates
Jones et al., 2015; USA (+15 countries)	Innocoll	RCT	Soft tissue only	NR	524	62	78	≤ 28	Local & systemic vs. systemic	Gentamycin‐collagen sponge 32.5‐130 mg OD with systemic antibiotics (NR)	Systemic antibiotics (NR)	Clinical cure; Reinfection rates, Amputation rates
Uckay et al., 2018; Switzerland	Innocoll	RCT	Soft tissue only	Moderate/Severe	88	71	64	14–28	Local & systemic vs. systemic	Gentamycin‐collagen sponge 32.5‐130 mg OD with systemic antibiotics	PO/IV of levofloxacin ± clindamicin OR amoxicillin‐clavulanate OR piperacillin‐tazobactam OR aztreonam OR metronidazole OR linezolid	Clinical cure; Clinical improvement; Complete microbiological eradication from baseline
Qin et al., 2019; China	None	Cohort	Osteomylitis	Moderate/Severe	46	62	61	42	Local & systemic vs. systemic	Gentamocin (80 mg)/vancomycin (0.5 g) impregnated calcium sulphate with IV antibioitcs (2 weeks) followed by oral antibioitcs (4 weeks) (NR)	IV antibioitcs (2 weeks) followed by oral antibioitcs (4 weeks) (NR)	Clinical cure; Time to clinical cure; Reinfection rates; Amputation rates
Alabdaly et al., 2019; Iraq	None	RCT	Soft tissue only	Mild/Moderate/Severe	60	NR	52	7–28	Local vs. systemic	Local solution of meropenine, cefipen or amikacin	PO/IV or meropenine, cefipen or amikacin	Clinical cure; Reinfection rates
Chatzipapas et al., 2020; Greece	None	Cohort	Osteomylitis	Moderate	25	64	72	7–38	Local & systemic vs. systemic	Gentamycin‐loaded PMMA beads OR gentamycin‐loaded hydroxyapatite and calcium sulfate beads with systemic antibiotics (NR)	Systemic antibiotics (NR)	Clinical cure; Time to clinical cure; Reinfection rates; Amputation rates
DeGiglio et al., 2021; Italy	None	Case–control	Osteomylitis	NR	44	68	68	≥ 14	Local & systemic vs. systemic	S53P4 glass with systemic antibiotics (NR)	Systemic antibiotics (NR)	Clinical cure; Time to clinical cure; Reinfection rates; Amputation rates
Brodell et al., 2021; USA	None	Cohort	Osteomylitis	NR	38	61	79	14–42	Local & systemic vs. systemic	Intraoperative vancomycin powder with systemic antibiotics (NR)	Systemic antibiotics (NR)	Amputation rates
Ehya et al., 2021; China	Natural Science Foundation of Hubei Province	RCT	Osteomylitis	Severe	36	47	67	NR	Local & systemic vs. systemic	Antibiotic‐laden PMMA bone cement (vancomycin, cefoperazone, orgentamicin) with IV antibioitcs (NR)	Vacuum sealed therapy with IV antibioitcs (NR)	Time to clinical cure; Reinfection rates; Complete microbiological eradication from baseline
Ding et al., 2022; China	Peking University	Cohort	Osteomylitis	Moderate/Severe	243	70	69	28–56	Local & systemic vs. systemic	Intraoperative gentamycin (OR vancomycin OR ceftazidine) bone cement 40 g STAT with systemic antibiotics (NR)	Systemic antibiotics (NR)	Time to clinical cure
Memon et al., 2022; Pakistan	None	Cohort	Soft tissue only	NR	140	46	62	?7	Local & systemic vs. systemic	10 g of Gentamycin 0.1% cream with ciprofloxacin 200 mg BD	Ciprofloxacin 200 mg BD	Clinical cure; Clinical improvement; Complete microbiological eradication from baseline
Ragghianti et al., 2023; Italy	None	Case–control	Osteomylitis	Severe	55	72	71	≤ 28	Local & systemic vs. systemic	Antibiotic‐impregnanted calcium sulphate (0.5 g vancomycin/120 mg tobramicin) with systemic antibioitcs (NR)	Systemic antibiotics (NR)	Clinical cure; Time to clinical cure; Reinfection rates; Amputation rates
Dai et al., 2023; China	Shanghai Municipal Health Commission	Cohort	Osteomylitis	Moderate/Severe	52	54	39	≥ 14	Local & systemic vs. systemic	Antibiotic‐laden PMMA bone cement (gentamicin & vancomycin) with systemic antibioitcs (NR)	Vacuum sealed therapy with IV antibioitcs (NR)	Clinical cure; Time to clinical cure; Amputation rates
Chow et al., 2024; Austraila	Unclear	Cohort	Osteomylitis	NR	136	59	62	NR	Local & systemic vs. systemic	Cerament G and/or V with endocrinology guided systemic antibioitcs	Systemic antibiotics (NR)	Amputation rates
Lipsky et al., 2024; USA	Microbion	RCT	Osteomylitis	Moderate/Severe	53	53	85	≥ 14	Local & systemic vs. systemic	Pravibismane 0.2–4 mL of 3/7.5/15 μg/cm^2^, 3× a week with systemic antibioitcs (NR)	Systemic antibiotics (NR)	Reinfection rates; Amputation rates

Abbreviations: BD, bis in die (twice daily); IV, intravenous; NR, not reported; OD, omne in die (once daily); PMMA, polymethyl methacrylate microspheres; PO, by mouth; RCT, randomized controlled trial; STAT, “now” single dose; USA, United States of America.

### 3.1 Risk of Bias Assessment

The overall risk of bias showed “some concern” to “high concern” for the randomized studies and “moderate” to “serious” concern for the included non‐randomized studies [Supplement 3]. This represents a lack of high‐quality, low bias studies in the field at present. It was determined that all studies should be included for meta‐analysis to accurately summarize the current state of evidence.

For randomized studies, most concerns related to the randomization process, which was often not detailed fully [15]. Included retrospective studies also demonstrated high levels of bias due to confounding; for example, one study documented that “Treatment strategy was at the discretion of a single experienced orthopaedic surgeon. There were no predefined criteria or randomization to the 3 groups” [20]. Bias in the measurement of outcome was also significant, particularly in non‐blinded RCTs and retrospective studies, where assessors of outcome were aware of participants' treatment group [25, 26, 39]. Studies that lacked a placebo also scored poorly, given the nature of comparison between topical and systemic therapy, where even blinded participants and outcome assessors would be aware which arm of the trial they were in [21, 22, 25, 26, 39].

Four studies were funded by Innocoll who manufacture the gentamicin sponge used in those studies [22, 23, 25, 26], one by both Magainin Pharmaceuticals and SmithKline Beecham [19], one by Microbion [28], and two by Oculus Innovative Sciences who manufacture Microcyn Rx and Dermacyn [24, 30]. The remaining were funded by academic organizations or self‐funded.

### 3.2 Clinical Cure

**FIGURE 3:**
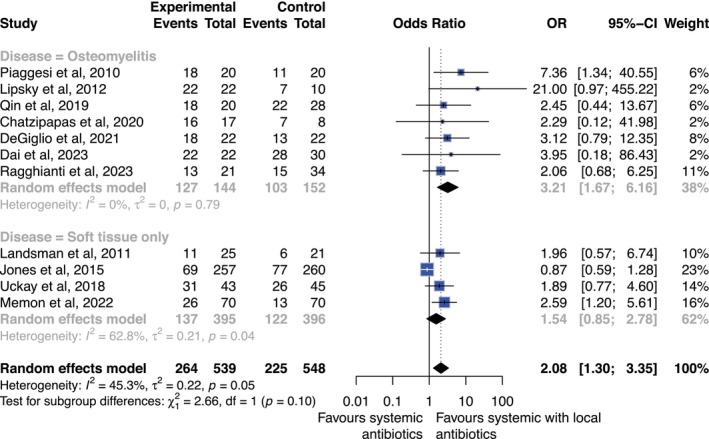
Forest plot diagram showing result of meta‐analysis for outcome clinical cure rates for combination versus systemic antibiotics. Sub‐analysis by soft tissue and bone disease. Results presented as odds ratio (OR) and 95% confidence intervals (CI).

There were 13 studies reporting on clinical cure rates including 1237 participants (1087 for combination and 150 for local only) [20, 21, 22, 23, 24, 25, 26, 30, 32, 36, 37, 38, 39]. Overall, combination therapy (local plus systemic therapy) was associated with a significant increase in cure rates versus systemic therapy alone (OR 2.08; 95% CI 1.30–3.35; *p* < 0.05); however, after sensitivity analysis, cure rates did not appear to differ and GRADE quality of evidence increased (OR 2.08; 95% CI 0.90–4.81; *p* = 0.09) [Table 2, Figure 3, Supplement 4].

**TABLE 2:** Table showing results of meta‐analysis for each outcome for combination (experimental) vs. systemic (control) only antibiotic therapy, with subgroup analysis by soft tissue and bone disease.

Outcome	Studies	Experimental		Control		Effect size	95% CI	*p* < 0.05	GRADE
		Events	Total	Events	Total				
*Clinical cure*									
Osteomyelitis	7	127	144	103	152	3.21	1.67–6.16	Y	—
Soft tissue	4	137	395	122	396	1.54	0.85–2.78	—	—
Overall	11	264	539	225	548	2.08	1.30–3.35	Y	Low
Overall (RCTs)	5	151	367	127	356	2.08	0.90–4.81	—	Moderate
*Clinical improvement*									
Osteomyelitis	1	19	21	10	16	5.70	0.97–22.69	—	—
Soft tissue	3	43	138	35	136	1.15	0.44–2.98	—	—
Overall	4	62	159	45	152	1.49	0.59–3.78	—	Very low
Overall (RCTs)	3	33	89	28	82	1.29	0.34–4.97	—	Very low
*Time to clinical cure* ^a^									
Osteomyelitis	8	—	270	—	278	−9.76	−15.10 to 4.42	Y	Very low
Osteomyelitis (no TTT)	7	—	142	—	163	−11.83	−15.75 to 7.90	Y	—
Osteomyelitis (RCTs)	2	—	40	—	41	−15.54	−23.17 to 7.91	Y	Moderate
*Infection recurrence*									
Osteomyelitis	7	6	155	39	137	0.14	0.05–0.34	Y	—
Soft tissue	1	11	257	6	260	1.89	0.69–5.20	—	—
Overall	8	17	412	45	397	0.23	0.08–0.71	Y	Moderate
Overall (RCTs)	4	15	334	21	331	0.32	0.06–1.64	—	Low
*Re‐intervention/amputation*									
Osteomyelitis	10	24	299	48	236	0.40	0.17–0.94	Y	—
Soft tissue	1	3	257	2	260	1.52	0.25–9.19	—	—
Overall	11	27	556	50	496	0.46	0.21–1.02	Y	Low
Overall (RCTs)	4	11	228	19	315	0.45	0.16–1.24	—	Low
*Complete microbiological eradication*									
Osteomyelitis	2	35	44	10	26	8.46	1.95–36.65	Y	—
Soft tissue	2	60	113	41	115	2.08	1.22–3.56	Y	—
Overall	4	95	157	51	141	2.54	1.55–4.17	Y	Low
Overall (RCTs)	3	61	87	30	71	4.33	1.19–15.76	Y	Low

*Note:* Sensitivity analysis performed using RCTs only for each outcome. Effects reported as OR, unless otherwise specified.

Abbreviation: TTT, tibia transverse transport.

^a^ Results reported as mean difference.

Three studies compared clinical cure rates of systemic only versus local only treatment for soft tissue DFI [21, 24, 26]. Meta‐analyses revealed that cure rate was similar between treatment groups [Table 3]. There was evidence of significant statistical heterogeneity between studies (*I* ^2^ = 66%) [Supplement 5].

**TABLE 3:** Table showing results of meta‐analysis for each outcome for local only (experimental) vs. systemic (control) only antibiotic therapy, with subgroup analysis by soft tissue and bone disease.

Outcome	Studies	Experimental		Control		OR	95% CI	*p* < 0.05	GRADE
		Events	Total	Events	Total				
*Clinical cure*									
Soft tissue (RCT only)	3	63	93	28	57	2.73	0.59–12.63	—	Very low
*Clinical improvement*									
Soft tissue (RCT only)	3	328	460	345	439	0.72	0.52–1.02	—	Low
*Infection recurrence*									
Soft tissue (RCT only)	2	29	459	22	432	0.56	0.06–5.48	—	Low
*Re‐intervention*									
Soft tissue (RCT only)	1	11	418	9	417	1.23	0.50–2.99	—	Very low
*Complete microbiological eradication*									
Soft tissue (RCT only)	2	147	349	159	343	0.84	0.62–1.14	—	Low

*Note:* Sensitivity analysis performed using RCTs only for each outcome. Effects reported as OR.

### 3.3 Clinical Improvement

Four studies compared combination antibiotic therapy to systemic antibiotics (311 total participants) and suggested that clinical improvement rates were similar between experimental and control groups (OR 1.49; 95% CI 0.59–3.78; *p* = 0.40) [Table 2, Supplement 5] [22, 24, 29, 39]. Uckay et al. also allowed a reduction in a customized “wound score” to quantify clinical improvement [22]. Similarly, three studies including 899 participants compared local antibiotics only with regards to clinical improvement and found that they were non‐inferior to systemic antibiotics alone (OR 0.72; 95% CI 0.52–1.02; *p* = 0.06) [Table 3, Supplement 5] [19, 24, 26].

### 3.4 Complete Microbiological Eradication From Baseline

Six studies with total 990 patients reported data on microbiological eradication [19, 22, 25, 26, 27, 39]. Treatment duration was 7 [39], 7–28 [25], 14–28 [19, 22], or 21–35 days [26]. Similarly, final follow up, which was also described as “test of cure” was between 35 and 42 days for all except Memon et al. where it was 7 days [39]. Sample sizes were generally small for studies looking at combination antibiotics (*n* = 36–140) [Table 1] [22, 25, 27, 39]. Overall, results for both osteomyelitis and soft tissue disease favored systemic and local antibiotics over systemic alone (OR 2.54; 95% CI 1.55–4.17; *p* < 0.05), and after sensitivity analysis similar results were achieved (OR 4.33; 95% CI1.19–15.76; *p* < 0.05) [Table 2, Supplement 5]. With regards to local antibiotics alone versus systemic antibiotic therapy, Prior et al. and Lipsky et al. concurred in their findings marginally favoring systemic antibiotics [Table 3, Supplement 5], however this was non‐significant on meta‐analysis (OR 0.84; 95% CI 0.62–1.14; *p* = 0.27) [19, 26].

### 3.5 Time to Clinical Cure

**FIGURE 4:**
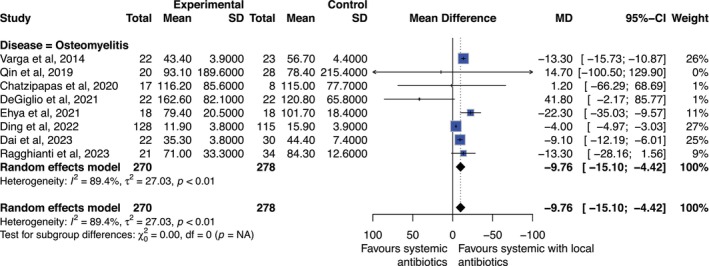
Forest plot diagram showing result of meta‐analysis for outcome time to clinical cure for combination versus systemic antibiotics. Results presented as mean difference (MD) in days and 95% confidence intervals (CI).

Eight studies including 548 participants reported on the time to clinical cure for combination antibiotics versus systemic antibiotics alone for DFI [20, 27, 31, 32, 33, 36, 37, 38]. There was evidence of statistical heterogeneity between included studies (*I* ^2^ = 89.4%). However, the random effects model showed that patients with combination antibiotics healed on average 9.8 days faster than those with systemic antibiotics alone (*p* < 0.05) [Table 2, Figure 4]. Time to healing varied greatly, especially in a study comparing tibia transverse transport (TTT) with and without antibiotic bone cement (ABC) [33], where healing time was much quicker (11.9 days); excluding this study from meta‐analysis did not affect the conclusion greatly (MD −11.8 days; 95% CI −15.8 to −7.9; *p* < 0.05) [33]. No studies assessed time to cure with local antibiotics only.

### 3.6 Infection Recurrence

Eight papers with 809 participants studied the effect of combination antibiotic therapy on infection recurrence rates (1 soft tissue only and 7 bone disease) [20, 23, 27, 28, 30, 32, 37, 38]. Meta‐analysis of included studies demonstrated that recurrence rates were lower with combination therapy (OR 0.23; 95% CI 0.08–0.71; *p* < 0.05), with a similar conclusion achieved following sensitivity analysis [Table 2, Supplement 5]. For local antibiotics versus systemic antibiotics, there were two studies with a total of 888 participants. Here experimental and control were not favored either way with similar recurrence rates (OR 0.56; 95% CI 0.06–5.48; *p* = 0.62), with statistical heterogeneity present (*I* ^2^ = 89%) [Table 3, Supplement 5].

### 3.7 Further Amputation or Debridement

Meta‐analysis of 11 papers (1052 participants) showed that combination antibiotics were non‐inferior to systemic alone regarding amputation rates (OR 0.46; 95% CI 0.21–1.02; *p* = 0.6) [Table 2, Supplement 5]. With regards to local antibiotics alone, there was only one study that included data relating to this outcome, with results suggesting systemic antibiotics non‐significantly reduced rates of amputation or need for further debridement in soft tissue disease (OR 1.23; 95% CI 0.50–2.99; *p* = 0.66) [Table 3, Supplement 5] [19].

## 4 Discussion

DFI remains a significant public health burden with lifetime prevalence between 19% and 34% in patients with diabetes, with significant morbidity and mortality and economic and social implications [3, 40]. Optimized antimicrobial therapy is central to preventing progression to osteomyelitis, limb‐threatening complications, and amputation [41]. Determining the most efficacious route of administration of antibiotics is essential to reduce these complications [5]. This review evaluated the role of locally applied antibiotics as either an alternative or adjunct to systemic therapy in DFI.

Overall, local antibiotics alone appeared non‐inferior to systemic therapy for clinical cure, with no clear evidence of harm. When combined with systemic antibiotics, local therapy demonstrated numerically higher cure rates, reaching statistical significance only in osteomyelitic disease (where the GRADE quality of evidence was low) [Table 2, Figure 3] [20, 21, 22, 23, 24, 25, 26, 32, 39]. These findings are consistent with prior evidence suggesting that local antibiotic delivery may enhance tissue penetration in poorly perfused diabetic wounds [42]. Similarly, pathological eradication was more frequently achieved with combination therapy; however, inconsistent correlation with clinical cure suggests that pathogen clearance alone may not determine infection resolution. Distinguishing infection from colonization remains challenging and possibly limits the clinical relevance of microbiological outcomes.

An important finding was the reduction in time to clinical cure, by nearly 10 days, with combination antibiotic therapy. Earlier resolution may reduce hospital length of stay, risk of hospital‐acquired infection, and rehabilitation burden. However, heterogeneity in study populations and interventions limits confidence in the magnitude of this effect [Table 2, Figure 4] [20, 32, 33]. For example, Ding et al. administered 40 g gentamicin bone cement and also performed TTT. This involves separating a window of tibial bone cortex and applying slight transverse traction with external fixation which has been shown to improve healing rates by theoretically promoting cellular metabolism, tissue regeneration, and re‐establishing microcirculation [33, 43]. This reduced the treatment time to only 11.9 days compared with the other studies included in the meta‐analysis [Table 2, Figure 4]. Removal of this study from the meta‐analysis, as part of sensitivity analysis, resulted in the same conclusion that combination antibiotics reduced time to healing (MD −11.8 days; 95% CI −15.8 to −7.9; *p* < 0.05) [Table 2, Supplement 5].

Topical antibiotics were administered in multiple formulations, including collagens, sponges, creams, beads, cement, powders, glass, and solutions, each with distinct release profiles and tissue penetration characteristics [44]. Formulations may offer benefits through elimination of dead space (sponges and solutions), local wound compression (cement, sponge, and glass), or controlling concentration of antibiotic distribution into surrounding tissues [45, 46]. Gentamicin beads offer sustained release over 2–6 weeks, while sponges typically offer concentrated release in the first 24 h [47, 48]. While orthopedic literature suggests sponges may offer advantages over beads, transferability to DFI is limited by differences in infection biology, biofilm behavior in prosthetic joints, and surgical requirements [45, 49]. As a result, this review was unable to identify a superior formulation for DFI, reflecting limited uptake and heterogeneous reporting in the available literature [3, 45, 46, 50].

The overall quality of evidence was relatively low. A major limitation was reliance on subjective outcome measures. Seven of 8 observational studies included demonstrated moderate concerns regarding outcome measurement, with one study employing an unvalidated “wound score” [22]. With the availability of validated wound scores such as Wound, Ischemia, and Foot Infection (WIfI), Wagner, and University of Texas classification systems, use of a custom tool reduces the validity of results [51]. Similarly, differences in the definition of recurrence, particularly with inclusion of those with previously clinically improved infection, are influenced by the subjective nature of clinical improvement; recurrence may have been recorded in cases where eradication was never completely achieved. As before, standardization of assessing clinical improvement, such as consensus on a specific DFI wound score, is required before accurate determination of infection recurrence between study groups is possible [52].

Although all studies excluded patients with vascular disease, the significant overlap of vascular and diabetic disease means clinical application of study findings must be carefully considered. Impaired perfusion affects both wound healing and systemic antibiotic delivery, and while local therapy may theoretically bypass these limitations, this hypothesis requires further validation [42]. Similarly, preceding surgical debridement was common but inconsistently applied and not required for study inclusion. Given its independent role in reducing bacterial load, debridement represents a significant confounder [53]. The impact of offloading and inpatient admission for participants receiving IV antibiotics was not commented on in any study and would also act as a confounder.

A moderate to high risk of bias in all included studies represented the limitations of the existing evidence base and further establishes a need for high‐quality randomized studies to confirm the role of local antibiotics in DFI. Here, sensitivity analyses restricted to randomized controlled trials attenuated several observed effects, indicating a reliance on lower‐quality observational data. Moreover, the role of funding and particularly industry involvement in provision of topical agents in several included studies raises concern regarding publication and observer bias [22, 23, 24, 25, 26].

Local application of antibiotics to wounds provides a direct route of administration, increasing wound penetration. There may be a place for these in outpatient settings or in mild disease, with practical advantages including reduced systemic side effects and not requiring systemic level monitoring. Combination antibiotic therapy was shown to offer some benefit to healing times and pathogen eradication in DFI but this did not translate into clinical cure, clinical improvement or reduced amputation rates when looking at randomized studies only.

This meta‐analysis indicates that the quality of evidence limits the ability to reach definitive conclusions due to high levels of bias and heterogeneity within included studies. Routine clinical adoption cannot be recommended without stronger evidence. Future research should prioritize high‐quality, prospective, and randomized trials with standardized inclusion criteria, validated wound assessment tools, and stratification by infection severity. Controlling for confounding interventions such as debridement, along with assessment of antimicrobial resistance and vascular status, will be essential. With validation, local antibiotic treatment may become a valuable addition to the multidisciplinary management of DFI, expanding therapeutic options for clinicians to combat this healthcare challenge.

## Author Contributions

A.K. and H.L.: Conceptualization, Data Curation, Formal analysis, Investigation, Methodology, Software, Visulaisation, Writing – Original Draft Preparation. H.M.: Data Curation, Investigation, Methodology, Writing – Review and Editing. M.E.: Conceptualization, Methodology, Supervision, Writing – Review and Editing. B.T.: Conceptualization, Methodology, Visualization, Writing – Review and Editing.

## Funding

The authors have nothing to report.

## Disclosure

The authors have nothing to report.

## Ethics Statement

The authors have nothing to report.

## Consent

The authors have nothing to report.

## Conflicts of Interest

The authors declare no conflicts of interest.

## Supporting information


**Data S1:** Supporting Information 1.

**Data S2:** Supporting Information 2.

## Data Availability

The data that supports the findings of this study are available in the Supporting Information of this article.
