# Supplementary material for: Local Versus Systemic Antibiotics for Diabetic Foot Infection—A Systematic Review and Meta‐Analysis
Source: J Diabetes. 2026 Mar 10;18(3):e70201. doi: 10.1111/1753-0407.70201 (PMC12973258; doi:10.1111/1753-0407.70201)
Supplement: Supplementary file 2 — Data S2: Supporting Information 2. [file JDB-18-e70201-s002.docx]

# Supplementary files

## Supplement 1

***Search strategy and terms*** – see attached spreadsheet

## Supplement 2

***Table showing inclusion and exclusion criteria for studies in thus review***

| **Subject** | **Included** | **Excluded** |
| --- | --- | --- |
| *Populations* | Adults 18+ with confirmed DFI, soft tissue infection, OM  Allow patients with preceding surgery e.g. debridement, minor amputation or drainage | Children  Non-human/lab based |
| *Interventions* | Local antibiotic systems (any type)  Local + oral or intravenous (systemic) antibiotics | Intraoperative antibiotics  Herbal remedies  Antiseptics as listed by BNF: chlorhexidine, povidone-iodine, hydrogen peroxide, potassium permanganate.  Dressings listed by BNF: Honey, Iodine, Chlorhexidine, Silver (unless silver sulfadiazine), alginate, hydrogel and hydrocolloid |
| *Comparison* | Oral or IV antibiotics |  |
| *Outcomes* | Cure rate  Improvement rate  Time to wound healing  Amputation/debridement free time  Ulcer recurrence  Pathogen eradication |  |
| *Study designs* | Primary research of any quantitative comparative study design from published and grey literature  RCT/observational | Non-scientific reports, letters and correspondences.  Non-comparative study  Reviews  Abstracts  Poster proceedings  Case reports  Case series  Guidelines  Textbooks |
| *Language* | English | Any other language |
| *Publication dates* | Any date | On going trial with no published data |

## Supplement 3

***Quality assessment / risk of bias results for included randomised studies. The risk-of-bias (RoB2) tool was used and data presented using*** [***robvis***](https://www.riskofbias.info/welcome/robvis-visualization-tool) ***(visualization tool).***


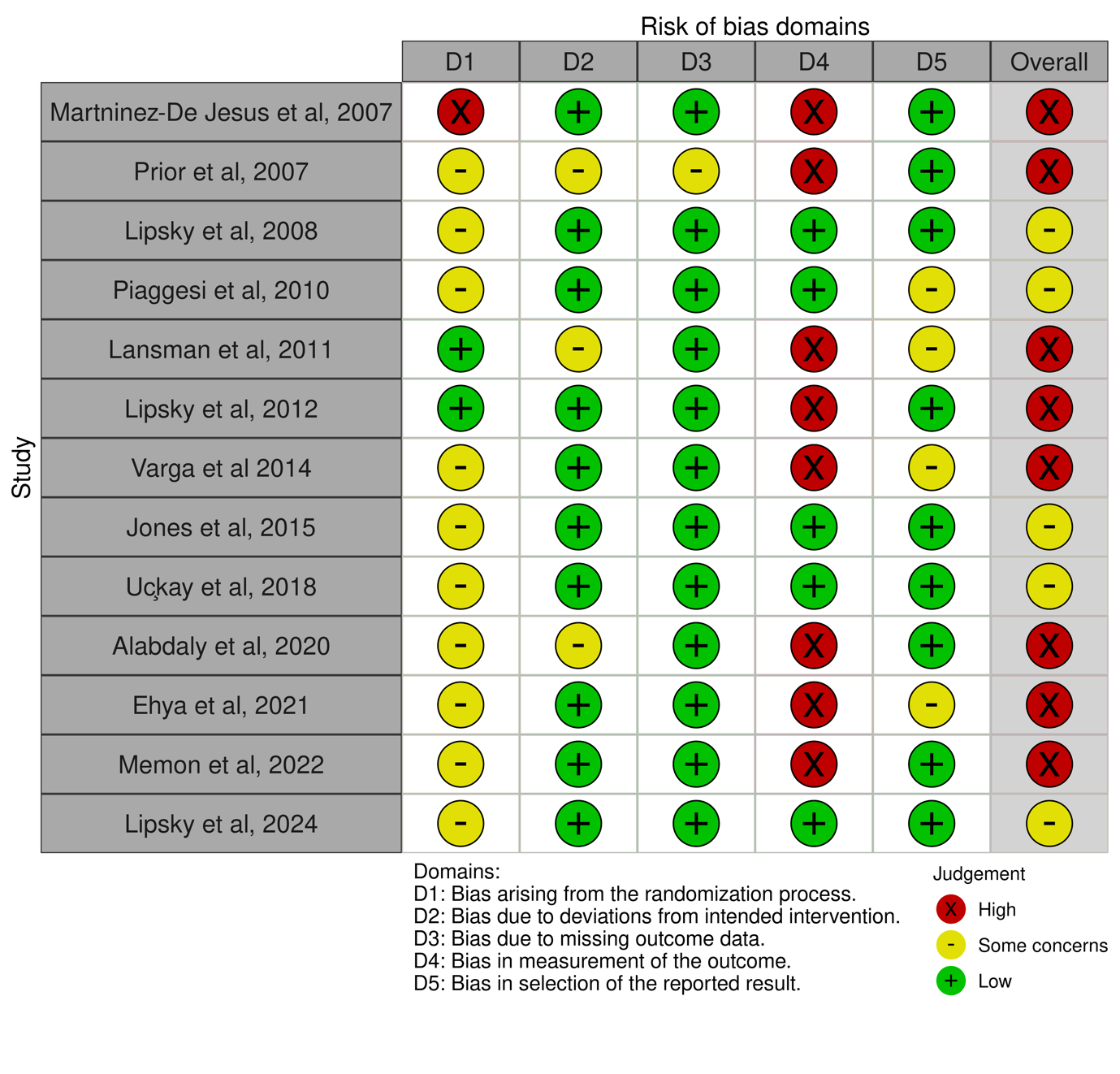


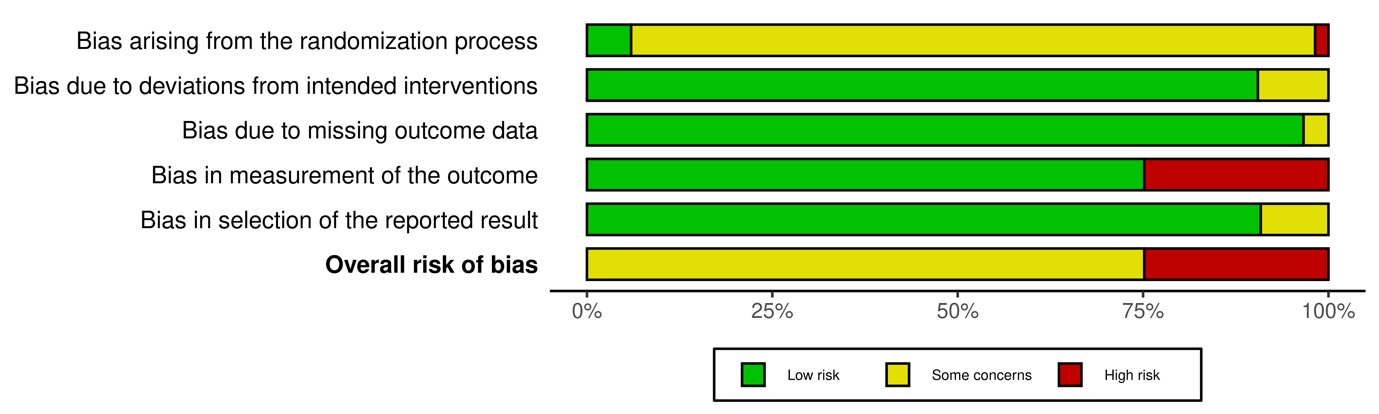


***Quality assessment / risk of bias results for included non-randomised studies. The Risk Of Bias In Non-randomised Studies - of Interventions (ROBINS-I) tool was used and data presented using*** [***robvis***](https://www.riskofbias.info/welcome/robvis-visualization-tool) ***(visualization tool).***


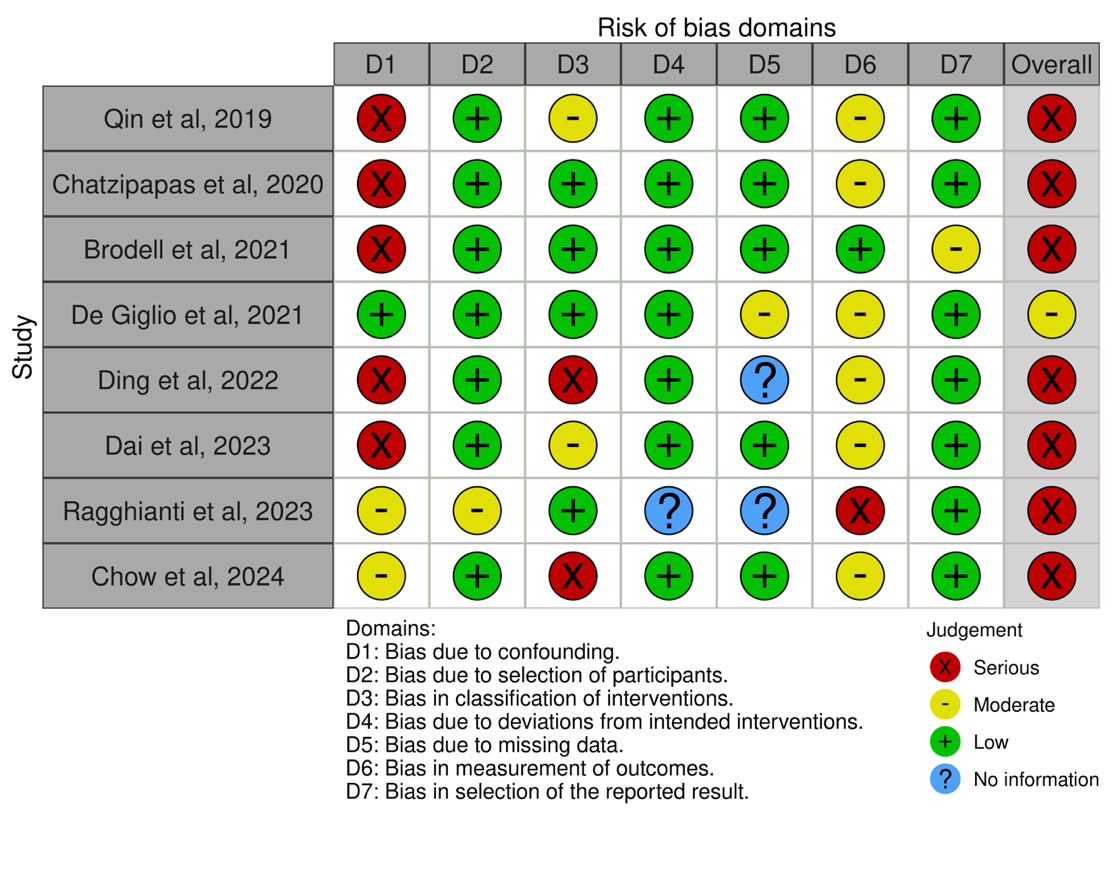


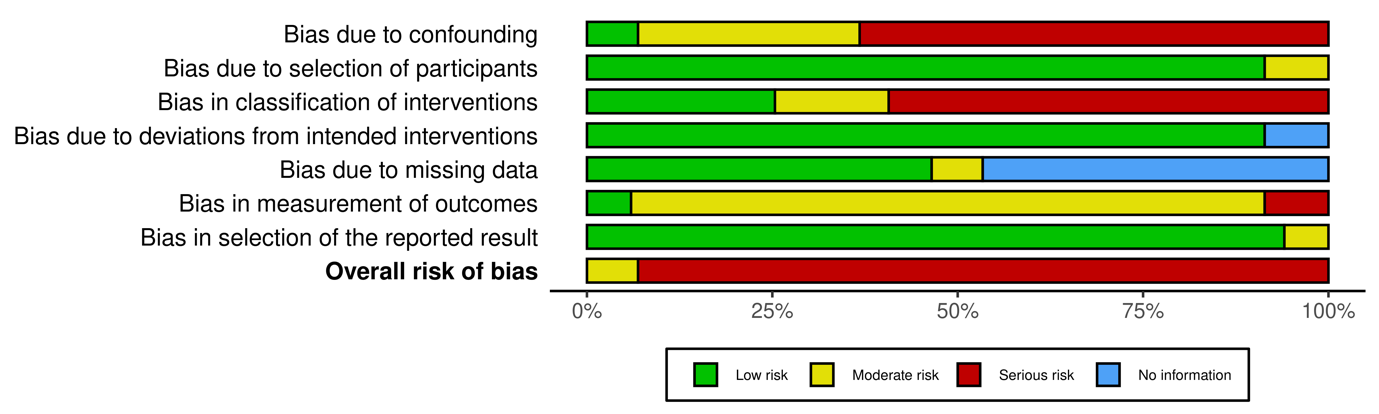


## Supplement 4

***GRADE Assessment results***

| **Summary of findings:** | | | | | | |
| --- | --- | --- | --- | --- | --- | --- |
| **Local antibiotics alone compared to systemic antibioitcs alone for diabetic foot infection** | | | | | | |
| **Patient or population:** diabetic foot infection  **Setting:** Vascular surgery  **Intervention:** local antibiotics alone  **Comparison:** systemic antibioitcs alone | | | | | | |
| Outcomes | **Anticipated absolute effects^*^** (95% CI) | | Relative effect (95% CI) | № of participants (studies) | Certainty of the evidence (GRADE) | Comments |
|  | **Risk with systemic antibioitcs alone** | **Risk with local antibiotics alone** |  |  |  |  |
| Clinical cure rate | 491 per 1,000 | **725 per 1,000** (363 to 924) | **OR 2.73** (0.59 to 12.63) | 150 (3 RCTs) | ⨁◯◯◯ Very low^a,b,c^ | The evidence is very uncertain about the effect of local antibiotics alone on clinical cure rate. |
| Clinical improvement rate | 786 per 1,000 | **725 per 1,000** (656 to 789) | **OR 0.72** (0.52 to 1.02) | 899 (3 RCTs) | ⨁⨁◯◯ Low^c,d^ | The evidence suggests that local antibiotics alone results in little to no difference in clinical improvement rate. |
| Infection recurrence rate | 51 per 1,000 | **29 per 1,000** (3 to 227) | **OR 0.56** (0.06 to 5.48) | 891 (2 RCTs) | ⨁⨁◯◯ Low^c,e^ | The evidence suggests that local antibiotics alone results in little to no difference in infection recurrence rate. |
| Re-intervention rate | 22 per 1,000 | **26 per 1,000** (11 to 62) | **OR 1.23** (0.50 to 2.99) | 835 (1 RCT) | ⨁◯◯◯ Very low^c,f^ | The evidence is very uncertain about the effect of local antibiotics alone on re-intervention rate. |
| Complete microbiological eradication rate | 464 per 1,000 | **421 per 1,000** (349 to 496) | **OR 0.84** (0.62 to 1.14) | 692 (2 RCTs) | ⨁⨁◯◯ Low^b,c^ | The evidence suggests that local antibiotics alone results in little to no difference in complete microbiological eradication rate. |
| ***The risk in the intervention group** (and its 95% confidence interval) is based on the assumed risk in the comparison group and the **relative effect** of the intervention (and its 95% CI).  **CI:** confidence interval; **OR:** odds ratio | | | | | | |
| **GRADE Working Group grades of evidence** **High certainty:** we are very confident that the true effect lies close to that of the estimate of the effect. **Moderate certainty:** we are moderately confident in the effect estimate: the true effect is likely to be close to the estimate of the effect, but there is a possibility that it is substantially different. **Low certainty:** our confidence in the effect estimate is limited: the true effect may be substantially different from the estimate of the effect. **Very low certainty:** we have very little confidence in the effect estimate: the true effect is likely to be substantially different from the estimate of effect. | | | | | | |

#### Explanations

a. Serious concerns in several studies

b. Small sample size

c. Several studies funded by industry

d. Bias in measurement of outcome

e. Heterogeneity

f. Only 1 RCT

| **Summary of findings:** | | | | | | |
| --- | --- | --- | --- | --- | --- | --- |
| **Combination (local & systemic) antibiotics compared to systemic antibiotics alone in diabetic foot infection** | | | | | | |
| **Patient or population:** diabetic foot infection  **Setting:** Vascular surgery  **Intervention:** combination (local & systemic) antibiotics  **Comparison:** systemic antibiotics alone | | | | | | |
| Outcomes | **Anticipated absolute effects^*^** (95% CI) | | Relative effect (95% CI) | № of participants (studies) | Certainty of the evidence (GRADE) | Comments |
|  | **Risk with systemic antibiotics alone** | **Risk with combination (local & systemic) antibiotics** |  |  |  |  |
| Clinical cure rate | 411 per 1,000 | **592 per 1,000** (475 to 700) | **OR 2.08** (1.30 to 3.35) | 1087 (11 non-randomised studies) | ⨁⨁◯◯ Low^a,b,c^ |  |
| Clinical cure rate (RCTs only) | 357 per 1,000 | **536 per 1,000** (333 to 727) | **OR 2.08** (0.90 to 4.81) | 723 (5 RCTs) | ⨁⨁⨁◯ Moderate^c,d^ | Combination (local & systemic) antibiotics probably results in little to no difference in clinical cure rate. |
| Clinical improvement rate | 317 per 1,000 | **409 per 1,000** (215 to 637) | **OR 1.49** (0.59 to 3.78) | 301 (4 non-randomised studies) | ⨁◯◯◯ Very low^a,b,e,f^ |  |
| Clinical improvement rate (RCTs only) | 341 per 1,000 | **401 per 1,000** (150 to 720) | **OR 1.29** (0.34 to 4.97) | 171 (3 RCTs) | ⨁◯◯◯ Very low^b,c,f,g^ | The evidence is very uncertain about the effect of combination (local & systemic) antibiotics on clinical improvement rate. |
| Time to clinical cure | The mean time to clinical cure was **53.72** days | MD **9.76 days fewer** (15.1 fewer to 4.42 fewer) | - | 548 (8 non-randomised studies) | ⨁◯◯◯ Very low^e,h,i^ |  |
| Time to clinical cure (RCTs only) | The mean time to clinical cure was **76.46** days | MD **15.54 days fewer** (23.17 fewer to 7.91 fewer) | - | 81 (2 RCTs) | ⨁⨁⨁◯ Moderate^a,f^ | Combination (local & systemic) antibiotics likely reduces time to clinical cure. |
| Infection recurrence rate | 113 per 1,000 | **29 per 1,000** (10 to 83) | **OR 0.23** (0.08 to 0.71) | 809 (8 non-randomised studies) | ⨁⨁⨁◯ Moderate^b^ |  |
| Infection recurrence rate (RCTs only) | 63 per 1,000 | **21 per 1,000** (4 to 100) | **OR 0.32** (0.06 to 1.64) | 665 (4 RCTs) | ⨁⨁◯◯ Low^b,c^ | Combination (local & systemic) antibiotics may result in little to no difference in infection recurrence rate. |
| Re-intervention rate | 101 per 1,000 | **49 per 1,000** (23 to 103) | **OR 0.46** (0.21 to 1.02) | 1052 (11 non-randomised studies) | ⨁⨁◯◯ Low^b,e^ |  |
| Re-intervention rate (RCTs only) | 60 per 1,000 | **28 per 1,000** (10 to 74) | **OR 0.45** (0.16 to 1.24) | 653 (4 RCTs) | ⨁⨁◯◯ Low^c,e^ | The evidence suggests combination (local & systemic) antibiotics reduces re-intervention slightly. |
| Complete microbiological eradication rate | 362 per 1,000 | **590 per 1,000** (468 to 703) | **OR 2.54** (1.55 to 4.17) | 298 (4 non-randomised studies) | ⨁⨁◯◯ Low^b,c^ |  |
| Complete microbiological eradication rate (RCTs only) | 423 per 1,000 | **760 per 1,000** (465 to 920) | **OR 4.33** (1.19 to 15.76) | 158 (3 RCTs) | ⨁⨁◯◯ Low^b,c^ | Combination (local & systemic) antibiotics may increase complete microbiological eradication slightly. |
| ***The risk in the intervention group** (and its 95% confidence interval) is based on the assumed risk in the comparison group and the **relative effect** of the intervention (and its 95% CI).  **CI:** confidence interval; **MD:** mean difference; **OR:** odds ratio | | | | | | |
| **GRADE Working Group grades of evidence** **High certainty:** we are very confident that the true effect lies close to that of the estimate of the effect. **Moderate certainty:** we are moderately confident in the effect estimate: the true effect is likely to be close to the estimate of the effect, but there is a possibility that it is substantially different. **Low certainty:** our confidence in the effect estimate is limited: the true effect may be substantially different from the estimate of the effect. **Very low certainty:** we have very little confidence in the effect estimate: the true effect is likely to be substantially different from the estimate of effect. | | | | | | |

#### Explanations

a. Bias in measurement of outcome

b. Serious concerns in several studies

c. Several studies funded by industry

d. Randomisation process not fully explained in 2 studies

e. Disease severity not reported in several studies

f. Small sample size

g. Variation in disease severity

h. High risk of bias from several studies due to confounding

i. Heterogeneity

# Supplement 5

Combination – Cure rates

Local – cure rates

Combination – clinical improvement

Local – clinical improvement

Combination – time to cure

Combination – time to cure (excluding Ding et al.)


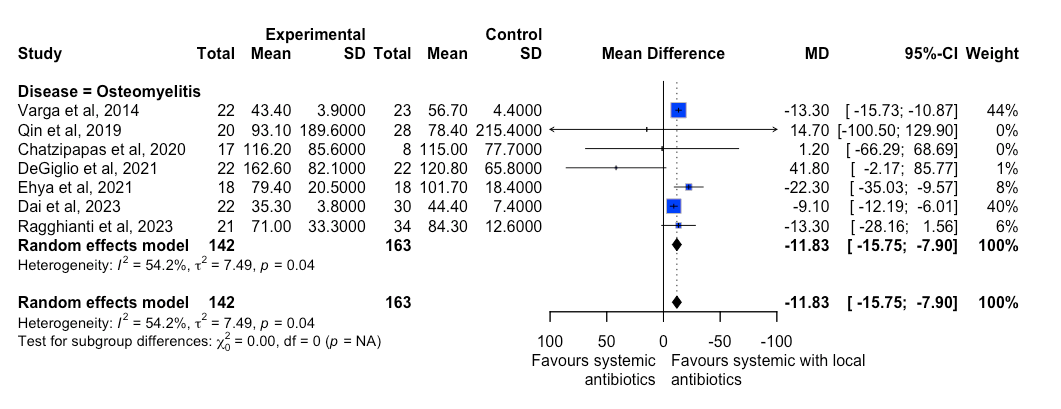


Combination – recurrence

Local – recurrence

Combination – reintervention

Local – reintervention

Combination – eradication

Local – eradication
